# Prediction of lung metastases in thyroid cancer using machine learning based on SEER database

**DOI:** 10.1002/cam4.4617

**Published:** 2022-02-22

**Authors:** Wenfei Liu, Shoufei Wang, Ziheng Ye, Peipei Xu, Xiaotian Xia, Minggao Guo

**Affiliations:** ^1^ Department of Thyroid, Parathyroid, Breast and Hernia Surgery Shanghai Jiao Tong University Affiliated Sixth People's Hospital Shanghai China

**Keywords:** lung metastasis, machine learning, partial dependency plot, prediction, thyroid cancer

## Abstract

**Purpose:**

Lung metastasis (LM) is one of the most frequent distant metastases of thyroid cancer (TC). This study aimed to develop a machine learning algorithm model to predict lung metastasis of thyroid cancer for providing relative information in clinical decision‐making.

**Methods:**

Data comprising of demographic and clinicopathological characteristics of patients with thyroid cancer were extracted from the National Institutes of Health (NIH)’s Surveillance, Epidemiology, and End Results (SEER) database between 2010 and 2015, which is employed to develop six machine learning algorithm models support vector machine (SVM), logistic regression (LR), eXtreme gradient boosting (XGBoost), decision tree (DT), random forest (RF), and k‐nearest neighbor (KNN). Compared and evaluated models by the following indicators: accuracy, precision, recall rate, F1‐score, the area under the ROC curve (AUC) value and Brier score, and interpreted the association between clinicopathological characteristics and target variables based on the best model.

**Results:**

Nine thousand nine hundred and fifty patients were selected, which including 212 patients (2.1%) with lung metastasis, and 9738 patients without lung metastasis (97.9%). Multivariate logistic regression showed that age, T stage, N stage, and histological type were independent factors in TC with LM. Evaluation indicators of the best model‐ RF were as following: accuracy (0.99), recall rate (0.88), precision (0.61), F1‐score (0.72), AUC value (0.99), and the Brier score (0.016).

**Conclusion:**

RF learning model performed better and can be applied to forecast lung metastasis of thyroid cancer, and offer valuable and significant reference for clinicians' decision‐making in advance.

## Graphical abstract

We used six machine learning models to identify the application of machine learning at prediction of the lung metastases in thyroid cancer patients based on SEER database.

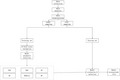

## 1 INTRODUCTION

Thyroid cancer (TC) is one of the most prevalent malignant tumors of the endocrine system, accounting for approximately 1%–3% of all new malignant tumors worldwide. Moreover, the occurrence of TC continues to increase in the USA.^1^, ^2^, ^3^ TC usually encompasses four histological types: papillary thyroid carcinoma (PTC), follicular thyroid carcinoma (FTC), medullary thyroid carcinoma (MTC), and anaplastic thyroid cancer (ATC).^4^ Therefore, TC generally exhibits an extensive range of clinical behavior, from indolent carcinomas with high survival rates to extremely aggressive malignancies, such as ATC, with high mortality rates. Hence, the prognosis of patients with TC also exhibits significant variability.^5^, ^6^ Generally, tumor metastasis greatly worsens the patient's prognosis and may even be the major factor contributing to the death of the patient. For differentiated TC, the most prevalent site of distant metastasis was the lung, which accounted for 85.6% of all distant metastases.^4^, ^7^, ^8^ Computerized tomography (CT) scans accurately detect lung metastasis (LM) in TC.^4^ However, it is well known that CT scans are ineffective in filtering out TC patients with a high risk of LM. Thus, the development of a clinical algorithm model for the prediction of LM in TC is beneficial in making medical decisions for diagnosis and treatment in advance to greatly improve patient prognosis. Over the years, advances in clinical models have reached a mature stage. There are perfect clinical models with high accuracy to predict the performance of malignant tumors, including nomograms forecasting survival in patients with ATC, radiomics nomogram for preoperative prediction of lymph node metastasis in colorectal cancer, and an individualized nomogram to identify occult peritoneal metastasis in patients with advanced gastric cancer.^9^, ^10^, ^11^

Generally, one topic in artificial intelligence is machine learning (ML), which primarily involves the exploration of the mechanism through which computers study data and the advancement algorithm model of learning procedures.^12^ ML are being utilized to address increasingly complex problems with astonishing success, particularly extensively applied in the medicine.^13^ Several studies have investigated the medical applications of machine learning, including medical image recognition, treatment support, and biomedical research.^14^, ^15^, ^16^

The surveillance, epidemiology, and end results (SEER) program is a database produced by the National Cancer Institute that provides data on cancer‐related incidence, stage, treatment, and patient survival rates. The database contains information from 18 population‐based tumor registries, having one nonrandom sample of 28% of the USA population, and records nearly 100% of the cancer cases in each registry.^17^

In the present study, our aim was to develop six machine learning algorithm models for predicting LM based on the SEER database and to compare the assessment indicators of models to select the optimal machine learning model for analyzing the correlation between LM and clinicopathological characteristics in patients with TC.

## 2 METHODS

### 2.1 Research idea

**FIGURE 1:**
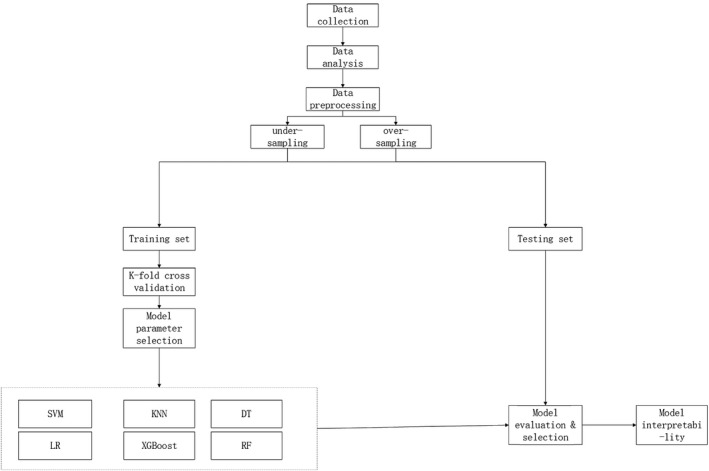
Research flow chart

This retrospective study utilized information from the SEER database to construct a binary classifier for predicting LM in patients with TC. The entire architecture process is illustrated in Figure 1.

### 2.2 Data collections

**FIGURE 2:**
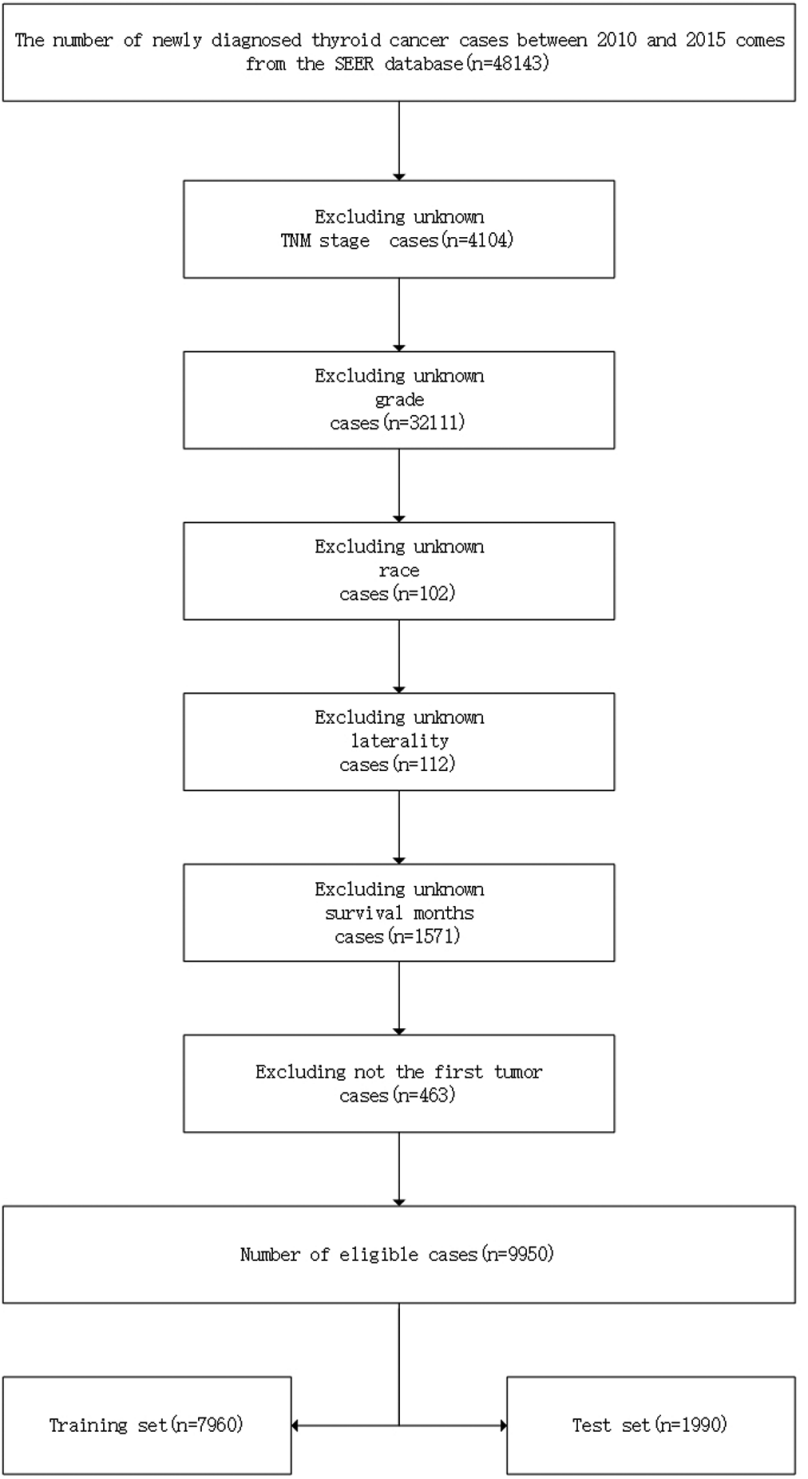
Detailed screening process of data collection

For this study, the following applicable data can be accessed from the SEER database after receiving SEER approval and permission. The following demographic and clinicopathological information of patients with TC from 2010 to 2015 was included: grade (grade I, well‐differentiated type; grade II, moderately differentiated type; grade III, poorly differentiated type; grade IV, undifferentiated type), T stage (T1a, T1b, T2, T3, T4a, and T4b), N stage (N0, N1a, and N1b), age, sex (male or female), race (White, Black, and others), laterality (solitary and multifocal), year of diagnosis, histological type [(PTC (8050, 8260), FTC (8330, 8331, 8332, 8335, 8337), MTC (8510) and ATC (8020, 8021, 8022)], and LM (yes, no). TNM staging is based on the 7th edition of the AJCC staging manual, and the histological type code refers to the ICD‐O‐3 manual.^18^ The following demographic and clinicopathological information of patients with TC from 2010 to 2015 was excluded: variables including TNM stage, grade, race, laterality, and survival months were unknown and not the first tumor. The detailed screening process is shown in Figure 2.

### 2.3 Analysis of information

The cases in question were separated into two groups: One category was LM, and the other category was NLM. Pearson's chi‐square test was used to compare the differences in clinicopathological characteristics between the two groups. A *p*‐value less than 0.05 demonstrates that the identical attributes differ significantly in the two groups of cases. Additionally, univariate logistic regression was performed to identify which characteristics features were closely associated with lung metastasis. Then, variables with univariate *p* value below 0.05 were considered for logistic multivariate analysis.

### 2.4 Data transformation

Research data were divided into feature variables including grade, T stage, N stage, age, gender, race, laterality, year of diagnosis, histological type, and target variable including LM.

One‐hot encoding for categorical variables includes T stage, N stage, gender, race, laterality, year of diagnosis, histological type, and sex. For instance, grade features with four values can be described as [(1000, 0100, 0010, 0001)].^19^

### 2.5 Sampling precession

**FIGURE 3:**
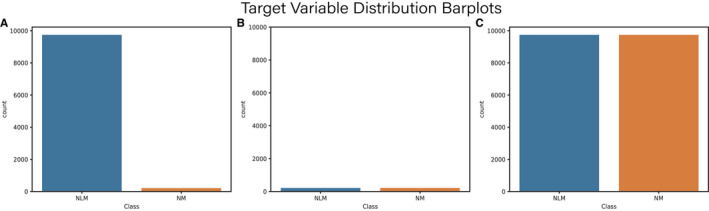
Target variable distribution of original data (A), under‐sampling data (B), and over‐sampling data (C)

**FIGURE 4:**
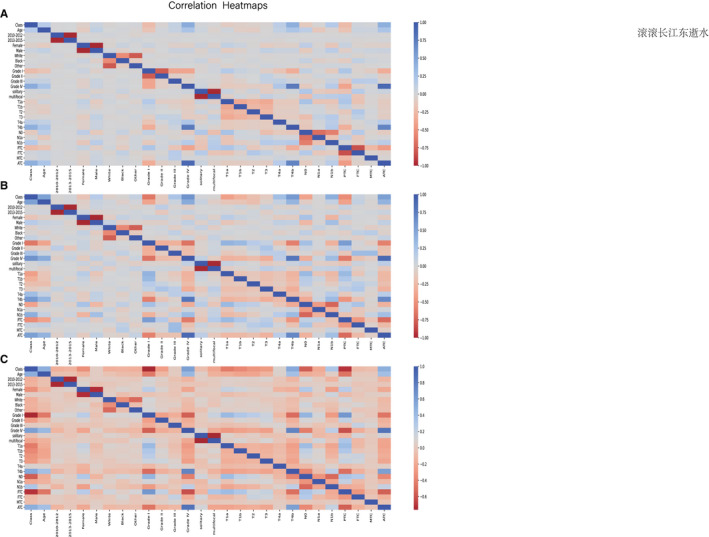
Correlation heatmaps of patients' characteristics features in original data (A), under‐sampling data (B), and over‐sampling (C)

Synthetic minority over‐sampling technique (SMOTE) or under‐sampling, a standard approach to balance classes on imbalanced datasets, is utilized to optimize the models.^28^ The distribution of the target variables after the sampling process as depicted in Figure 3. Meanwhile, the correlation between variables is clearer, as illustrated in Figure 4.

### 2.6 Data sampling

Although LM of TC is one of the most frequent distant metastases, the incidence of TC metastasis in all patients with TC is extremely low. Hence, it is evident that the original dataset is an extremely unbalanced dataset. Therefore, we adopt low‐sampling and over‐sampling techniques to address the original data and use the correlation matrix to analyze the alternation in the original data after sampling. The data after the sampling process were split into a training set (80%) and a test set (20%).

### 2.7 Model developments

The training set was used to develop six machine learning models, including support vector machine (SVM), logistic regression (LR), extreme gradient boosting (XGBoost), decision tree (DT), random forest (RF), and k‐nearest neighbor (KNN). The SVM is a binary classifier that is typically applied to precisely divide something with multidimensional attributes into two categories based on hyperspace.^20^ The LR model is used to study the impact of trait variables on the target variable, which is usually a binary classifier, such as the presence or absence of LM in patients with TC.^21^ DT models can accurately identify seven tumor histopathologies with a high classification rate.^22^ XGBoost, previously used to predict the association of miRNA diseases, is a machine learning algorithm implemented under the gradient boosting framework.^23^ The RF, which can be used to decrease training variance and improve integration and generalization, refers to a machine learning classifier that uses multiple trees to train and predict samples.^19^ The KNN is one of the most widely used nonparametric classification methods, which is based on the belief that if most of the k‐nearest samples in the vicinity of a sample belong to a specific class in the feature space, the sample also belongs to this category.^24^ Fivefold cross‐validation and parameter selection methods were adopted for model optimization.

### 2.8 Model tests and evaluation

The test set was used to detect six machine learning models. The indicators, comprising accuracy, precision, recall rate, F1_score, area under the ROC curve (AUC) value, and Brier score, were used to evaluate six machine learning models. The AUC value, calculated based on the ROC curve, which is a graphical plot showing the diagnostic capability of a binary classifier as its discrimination threshold is changed, is a standard indicator in the model evaluation. The Brier score is a proper score function that quantifies the accuracy of probabilistic predictions. It is applied to tasks in which predictions must assign probabilities to a set of mutually exclusive discrete outcomes. The closer the score is to zero, the more accurate the model.^25^

### 2.9 Model interpretability

Considering the perfect application of artificial intelligence in medicine, an intuitive interpretation of the machine learning model and the confirmation of the practical meaning of the model is essential. Target variable distribution graphs were plotted to illustrate the original distribution of the target variable relative to the feature variables. Partial dependency plots (PDPs) were created to illustrate the overall distribution of the target variable by the feature variables and the effect of the feature variables on the response of the target variable.^26^, ^27^ We evaluated the model by comparing the tendency of target variables to change with respect to the feature variables in the actual situation and that in the model prediction.

### 2.10 Data analysis software

Software including R 3.8.9 (https://www.r‐project.org/), Python 3.8.0 (https://www.python.org/), and SEER*Stat (https://seer.cancer.gov/seerstat/) were used in this study. The used packages were shown in Table 1.

**TABLE 1:** Detailed information about the packages used in the development of machine learning models

Package names	Version	Description
Numpy	1.19.5	Numpy is the fundamental package for array computing with python
Pandas	1.0.4	Powerful data structures for data analysis, time series, and statistics
Matplotlib	3.3.2	Python plotting package
Sklearn	0.0	A set of python modules for machine learning and data mining
XGBoost	1.2.0	XGBoost python package
Imblearn	0.0	Toolbox for imbalanced dataset in machine learning
PDPbox	0.2.1	Python partial dependence plot toolbox

## 3 RESULTS

### 3.1 Analysis of information on TC patients

A total of 9950 cases with TC were available, including 212 (2.1%) cases with LM and 9738 (97.9%) cases without LM. Comparing the two groups, the mean age of LM patients was significantly higher than that of NLM patients (64.52 ± 14.73 years vs. 46.87 ± 15.54 years; *p* < 0.001). Apart from the year of diagnosis, laterality, and race, the remaining feature variables were significantly different between the two groups (all *p* < 0.001), including gender, grade, T stage, N stage, and histological type. Detailed information is summarized in Tables 2 and 3. Univariate logistic analysis showed that age, sex, grade, T stage, N stage, and histological type were significant with LM as illustrated in Table 4. Multivariate logistic regression showed that all these variables, except sex, were independently related with LM (Table 5).

**TABLE 2:** The detailed demographic information of the patients with thyroid cancer

Categories	NLM [*n* (%)]	LM [*n* (%)]	*p* value
*n*	9738 (97.9)	212 (2.1)	*n*
Year of diagnosis			0.894
2010–2012	5122 (52.6)	113 (53.3)	
2013–2015	4616 (47.4)	99 (46.7)	
^a^Age(years)	46.87 ± 15.54	64.52 ± 14.73	<0.001
Sex			<0.001
Male	2438 (25.0)	92 (43,4)	
Female	7300 (75.0)	120 (56.6)	
Race			0.149
White	7765 (79.7)	158 (74.5)	
Black	651 (6.7)	16 (7.5)	
Others	1322 (13.6)	38 (17.9)	
Year of diagnosis			0.894

Abbreviations: LM, lung metastasis; NLM, none lung metastasis.

^a^ Mean values ± Standard Deviation.

**TABLE 3:** The detailed pathological characteristics of the patients with thyroid cancer

Categories	NLM [*n* (%)]	LM [*n* (%)]	*p* value
*n*	9738 (97.9)	212 (2.1)	
Laterality			
Solitary	5929 (60.9)	138 (65.1)	0.241
Multifocal	3809 (39.1)	74 (34.9)	
Grade			
Grade I	7769 (79.8)	35 (16.5)	<0.001
Grade II	1395 (14.3)	15 (7.1)	
Grade III	322 (3.3)	34 (16.0)	
Grade IV	252 (2.6)	128 (60.4)	
T stage			
T1a	3014 (31.0)	2 (0.9)	<0.001
T1b	2286 (23.5)	7 (3.3)	
T2	1695 (17.4)	8 (3.8)	
T3	2186 (22.4)	32 (15.1)	
T4a	306 (3.1)	43 (20.3)	
T4b	251 (2.6)	120 (56.6)	
N stage			
N0	6819 (70.0)	66 (31.1)	<0.001
N1a	1677 (17.2)	32 (15.1)	
N1b	1242 (12.8)	114 (53.8)	
Histological type			
PTC	8474 (87.0)	68 (32.1)	<0.001
FTC	975 (10.0)	33 (15.6)	
MTC	99 (1.0)	5 (2.4)	
ATC	190 (2.0)	106 (50.0)	

Abbreviations: ATC, anaplastic thyroid cancer; FTC, follicular thyroid cancer; MTC, medullary thyroid cancer; NLM, none lung metastasis; PTC, papillary thyroid cancer.

**TABLE 4:** Univariate analysis of variables related to central lung metastasis (LM)

Variables	OR	95%CI	*p* value
Year of diagnosis			
2010–2012	Reference		
2013–2015	1.029	0.783–1.351	0.8391
^a^Age(years)	1.076	1.066–1.086	<0.001
Sex			
Male	2.296	1.743–3.024	<0.001
Female	Reference		
Laterality			
Solitary	1.198	0.901–1.594	0.2145
Multifocal	Reference		
Race			
White	1.17	0.647–2.113	0.6039
Others	0.828	0.492–1.393	0.4769
Black	Reference		
Grade			
Grade I	Reference		
Grade II	2.387	1.3–4.382	0.005
Grade III	23.438	14.431–38.065	<0.001
Grade IV	112.747	76.005–167.252	<0.001
T stage			
T1a	Reference		
T1b	4.615	0.959–22.21	0.0565
T2	7.113	1.51–33.495	0.0131
T3	22.06	5.288–92.037	<0.001
T4a	211.768	51.114–877.355	<0.001
T4b	720.477	177.296–2927.8	<0.001
N stage			
N0	Reference		
N1a	1.971	1.288–3.017	0.0018
N1b	9.483	6.962–12.918	<0.001
Histological type			
PTC	0.061	0.04–0.092	<0.001
FTC	0.072	0.026–0.202	<0.001
MTC	0.015	0.01–0.02	<0.001
ATC	Reference	Reference	Reference

Abbreviations: ATC, anaplastic thyroid cancer; CI, confidence interval; FTC, Follicular thyroid cancer; LM, lung metastasis; MTC, medullary Thyroid Cancer; NLM, none lung metastasis; OR, odds ratio; PTC, papillary thyroid cancer.

^a^ Mean continuous variable.

**TABLE 5:** Multivariate analysis of variables related to lung metastasis (LM)

Factors	OR	95% CI	*p* value
^a^Age(years)	1.027	1.015–1.038	<0.001
Sex			
Male	1.214	0.871–1.692	0.2514
Female	Reference		
Grade			
Grade I	Reference		
Grade II	1.48	0.792–2.766	0.2185
Grade III	4.523	2.49–8.214	<0.001
Grade IV	5.797	2.691–12.488	<0.001
T stage			
T1a	Reference		
T1b	3.865	0.8–18.677	0.0925
T2	4.076	0.85–19.54	0.0789
T3	8.459	1.974–36.242	0.004
T4a	28.037	6.305–124.668	<0.001
T4b	41.528	9.052–190.527	<0.001
N stage			
N0	Reference		
N1a	1.846	1.12–3.043	0.0163
N1b	3.95	2.66–5.865	<0.001
Histological type			
PTC	2.306	1.108–4.8	0.0254
FTC	0.492	0.147–1.645	0.2495
MTC	0.681	0.378–1.227	0.2011
ATC	Reference		

Abbreviations: ATC, anaplastic thyroid cancer; CI, confidence interval; FTC, follicular thyroid cancer; LM, lung metastasis; MTC, medullary thyroid cancer; NLM, none lung metastasis; OR, odds ratio; PTC, papillary thyroid cancer.

^a^ Mean continuous variable.

### 3.2 Model performances

**FIGURE 5:**
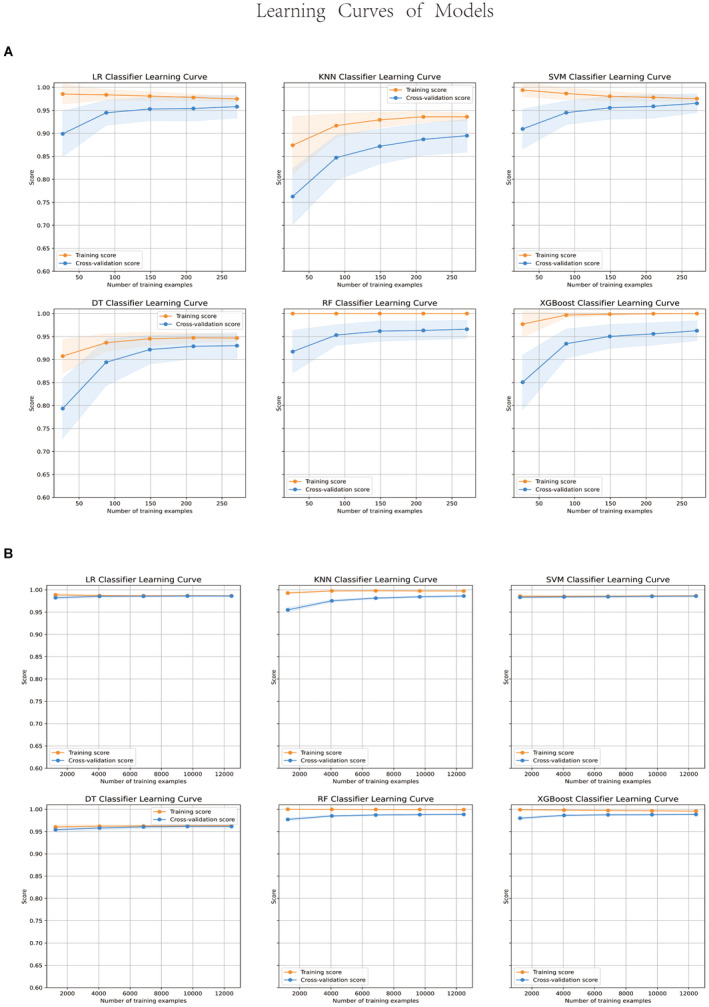
Learning curves of models with under‐sampling data (A) and over‐sampling (B)

**FIGURE 6:**
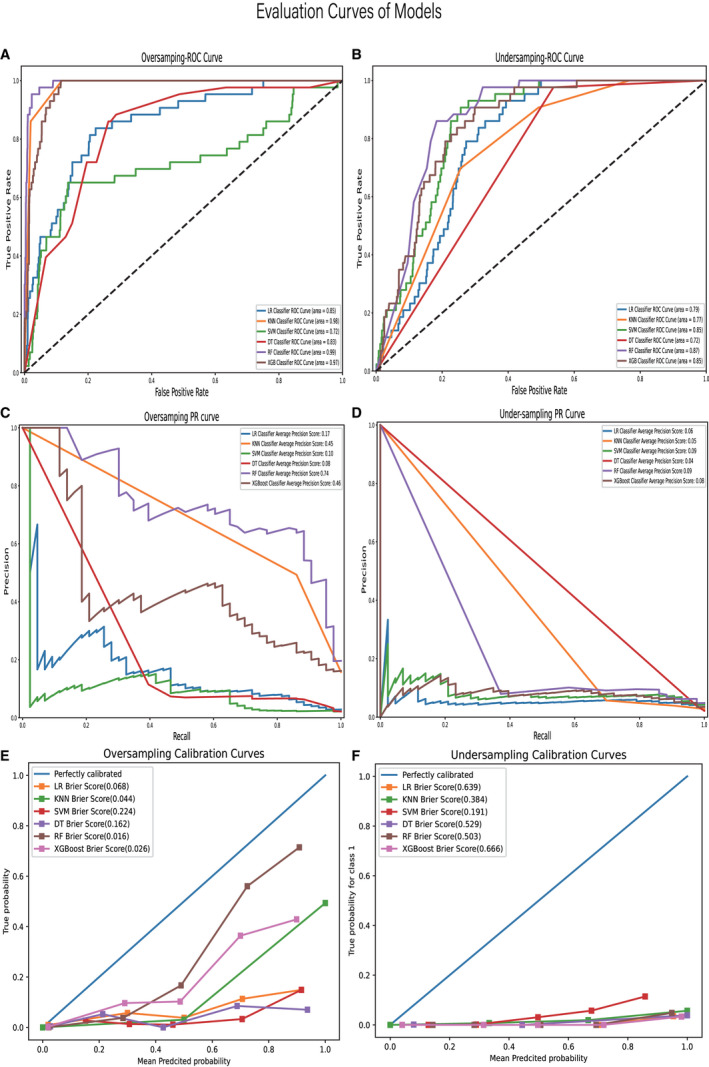
ROC curves of models developed by over‐sampling (A); ROC curves of models developed by under‐sampling (B); PR curves of models developed by over‐sampling (C); PR curves of models developed by under‐sampling (D); calibration curves of models developed by over‐sampling (E); calibration curves of models developed by under‐sampling (F)

Six machine learning models were developed and compared based on learning, receiver operating characteristic (ROC), precision‐recall (PR), and calibration curves. The machine learning model trained with the data processed by the over‐sampling method was better than that with the data processed by under‐sampling method. All learning curves are shown in Figure 5. The accuracy of all models was higher than 90%. However, the accuracy was not insufficient to explain the performance of the model owing to the imbalance of the dataset. The PR curve is desired to compensate for the shortcomings of the ROC curves and evaluate the advantages and disadvantages of the model. At 74%, the average precision of the RF model accuracy was significantly higher than those of the other models. Among these, the RF machine model performs better than the other machine learning models; the model with the highest accuracy (0.99), recall rate (0.88), precision (0.61), F1 score (0.72), and Brier Score (0.016). All evaluation curves are shown in Figure 6.

### 3.3 Interpretability of the model

**FIGURE 7:**
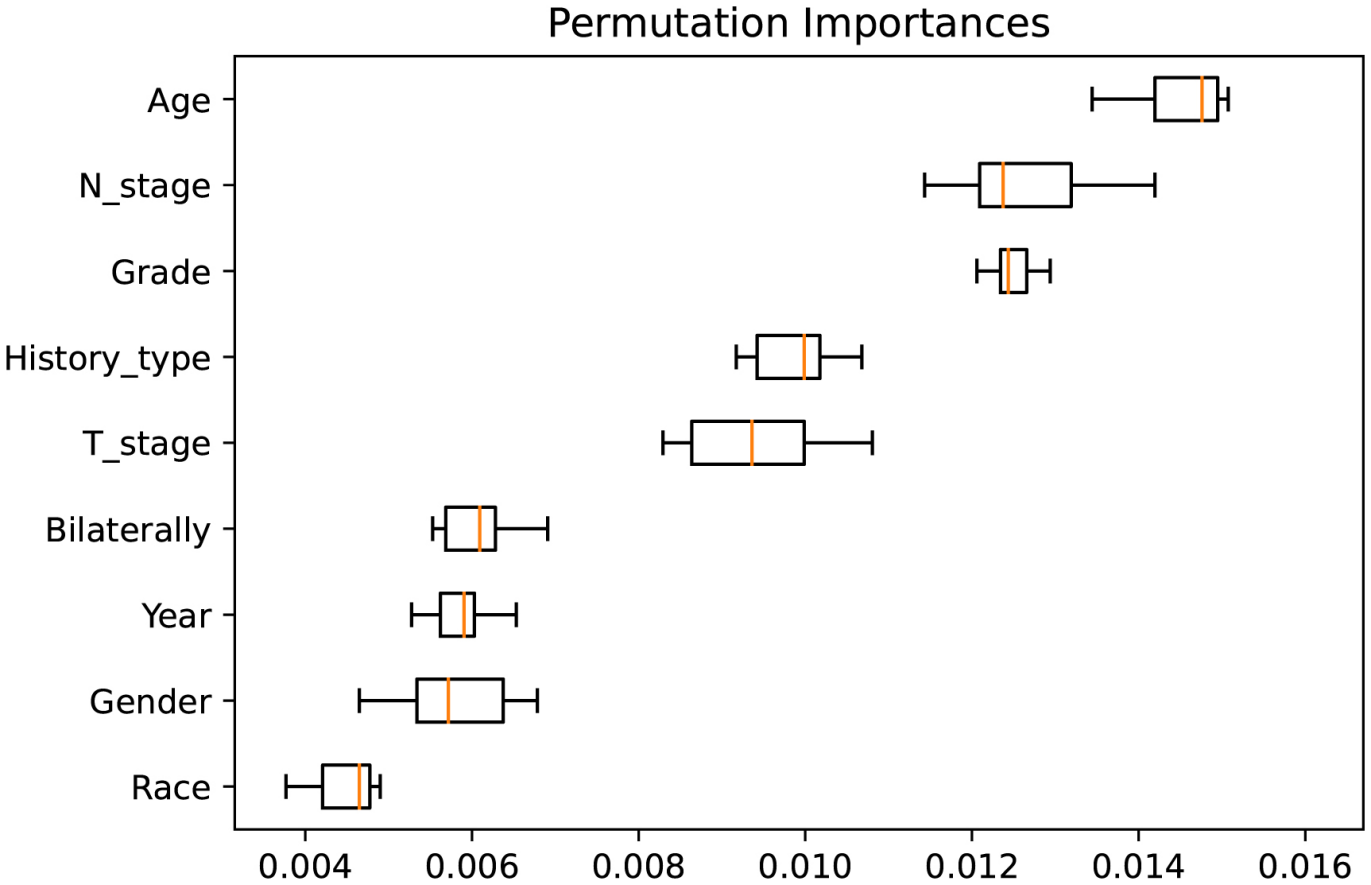
Importance ranking of feature variables

**FIGURE 8:**
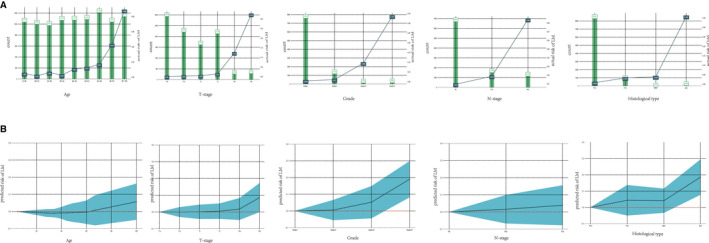
Actual risk of LM related to clinical characteristics (A) and the partial dependent plots of clinical characteristics (B) shaded part represents the confidence interval

T stage, grade, histological type, age, and N stage were critical to LM according to permutation importance based on the RF model in Figure 7. In terms of age, T stage, N stage, grade, and histological type, the actual risk change trend for LM agrees with the change trend in the risk of LM predicted by the model. The optimal predictive model shows that from 20 to 40 years of age, the risk of LMs decreases with increasing age. However, from the age of 60 years, the risk of LMs increases with age. The risk of LM increases gradually with an increase in the degree of T staging, N staging, and grade. Patients with PTC had the lowest risk of LM, those with FTC and ATC had nearly the same risk of LM, and those with ATC had the greatest risk of LM. Partial dependency diagrams of the five key traits are shown in Figure 8.

## 4 DISCUSSION

Six machine learning models consisting of SVM, XGBoost, LR, DT, RF, and KNN were designed to predict the LM in patients with TC based on the SEER database in the current retrospective research. Owing to the imbalance dataset, we not only utilize comprehensive scoring indicators, including accuracy, precision, recall rate, F1‐score, AUC value, and Brier score, but also over‐sampling and under‐sampling to improve model performance. In our research, we found that all models developed using over‐sampling processed original data were significantly better than low‐sampling, which demonstrates that the over‐sampling method is better suited for developing machine models for an extremely unbalanced dataset. A possible reason may be that the sample size of positive patients with LM deters the models to accurately identify the critical features of cases with LM. Ming Hao et al. also highlighted that the SOMTE algorithm can be broadly applied to solve an unbalanced classification problem in categorizing unbalanced PubChem BioAssay data, which is consistent with our findings.^29^ In addition, we found that although the accuracy of the model was higher than 90%, the precision of the model was not ideal, even below 50%. Hence, we believe that accuracy cannot be applied as the only model scoring indicator for models in unbalanced classification problems. We believe that because of the unbalanced data distribution in the classification problem, the models operate with false high accuracy. After applying the two processing methods of data sampling and multiple evaluation scores, model scoring indicators such as accuracy (0.99), precision (0.61), recall rate (0.88), F1score (0.71), and Brier score (0.016) proved that the RF model outperforms the other models. In the current study, the accuracy of the RF algorithm was unparalleled. First, the RF model is a type of ensemble learning algorithm with perfect advantages for processing massive amounts of data. Second, the RF model algorithm offers approaches to balance errors in unbalanced datasets. We believe that the accuracy of the RF algorithm is unsurpassed as it is a type of ensemble learning algorithm with perfect advantages for processing massive data and offers approaches to compensate for errors in unbalanced datasets in the current study.

In addition, the clinical and practical importance of machine learning lies in the detection of risk factors that are closely associated with LM. According to the permutation importance of feature variables, T stage, grade, histological type, age, and N stage were critical to LM. In a previous study, Li et al. demonstrated that T stage was an independent prognostic factor for the prognosis of patients with differentiated TC, which agrees with our study results.^30^ We found 212 cases of LM, accounting for 2.1% of the 9950 patients with TC. In addition, ATC patients were the most susceptible to LM, accounting for half of the total patients with LM, which suggests that ATC is a deadly and aggressive type of TC.^31^ Furthermore, grade and histological types are essential features of LM in machine learning models, further confirming the appealed view. Although CT is the most sensitive tool for the diagnosis of LM in TC, treatment is delayed when a patient with a high risk for LM is diagnosed with LM by CT scan.^32^, ^33^ Therefore, a machine learning model with the ability to predict the LM is required. Clinicians should focus on screening for medical intervention in disease development in patients with a high tendency for LM. Several studies have reported that age is an independent factor for the prognosis of TC patients. In general, the prognosis of younger patients with TC is better than that of older patients.^34^, ^35^ In the current study, we also discovered that age plays an essential role in TC patients with LM. We found that the N stage of TC patients is an influencing aspect of LM. Zhang et al. demonstrated that N1 patients were more likely to have LM than N0 patients with TC.^36^ In addition to explaining the ranking of the importance of feature variables, we interpret the effect of feature variables on the response of target variables using the PDP method first proposed by Friedman.^27^ We concluded that the probability of LM in TC patients gradually increased as the T stage level increased. Moreover, our research indicates that the LM risk increases sharply from T4a to T4b. In a previous study, Wang et al. reported that patients with earlier T stages exhibited significantly better overall survival and cancer‐specific survival in the univariate analysis.^37^ A possible reason for this is that the invasion of tumor cells into the prevertebral fascia, carotid artery, and mediastinal vessels accelerates the LM of tumor cells in patients with T4a or T4b stage; therefore, we observed that the likelihood of LM in Grade IV of TC is greater than 0.3, implying that undifferentiated TC is extremely like LM. Zhang et al. also demonstrated that undifferentiated TC was an independent prognostic factor for disease‐specific survival.^38^ We suspect that mutations in genes of including RAS, BRAF V600E, mTOR, NF1, NF2, MLH1, MLH3, MSH5, MSH6, ERBB2, EIF1AX, and USH2A were closely related to the overexpression of vascular endothelial growth factor to strongly promote LM in undifferentiated TC.^39^ We also found that as age increased, the likelihood of LM increased rapidly in TC patients over 60 years of age. In addition, a major change in the 8th AJCC staging system is that the age cutoff used for staging at diagnosis in TNM staging of differentiated TC changed from 45 to 55 years.^40^ In addition, we also noticed that people under the age of 60 have a reduced risk of LM from TC with age and a minimal risk of developing LM from the ages of 20 to 40 years. Therefore, the frequency of CT scans in TC patients older than 60 years should be higher than that of younger TC patients with earlier detection of LM. The N1b stage patients were more likely to have LM than those at N0 and N1a stages of TC, which is evident in the current study. Zhang et al. also proposed that N1 patients were more likely to have LM than N0 patients and that N1b stage patients had a higher risk of death.^36^ We believe that the most likely explanation is that lateral lymph node metastasis should be helpful for the migration of tumor cells to distant organs through lymphatic vessels.

### 4.1 Limitation and future improvement

This study aimed to develop six machine learning algorithm models to accurately predict LM in TC based on the SEER database. In addition, we visually presented the change trend and distribution of the LM relative to demographic and clinicopathological characteristics, and detailed the response of the target variable for each feature variable to overcome the unavailable explanation of models. However, there are some limitations in our study. First, the algorithm model is skewed because important medical information about molecular diagnosis, such as the BRAF gene mutation in TC patients, is not available. Second, it is difficult to apply the models to the population, as the evolution of the models is based on the data extracted from the SEER database in North America. Third, although the accuracy of the models was over 90%, prospective research is required to further verify the practice of the model. For LM diagnosis in TC, a complete system of artificial intelligence will be utilized in practice in the future, based on models of machine learning algorithms that significantly improve the prognosis of patients with advanced TC.

## 5 CONCLUSION

In this study, we developed six machine learning models to predict LM in patients with TC. All models performed well, and the RF model had a better predictive power. We also obtained clinical feature interpretations to provide clinicians with relative information for reference in clinical decision‐making.

## CONFLICT OF INTEREST

The authors report no declarations of interest.

## AUTHOR CONTRIBUTIONS

WF L, SF W, and MG G conceived of and designed the study. PP X, ZH Y, and XT X performed literature search. WF L generated the figures and Tables. SF W and PP X analyzed the data. WF L wrote the manuscript and MG G critically reviewed the manuscript. MG G supervised the research. All authors have read and approved the final manuscript.

## ETHICAL APPROVAL STATEMENT

We received permission to access the research data file in the SEER program from the National Cancer Institute, US. Approval was waived by the local ethics committee, as SEER data are publicly available and de‐identified.

## Data Availability

The datasets generated during and/or analyzed during the current study are available from the corresponding author on reasonable request
